# Editorial: Women in autoimmune and autoinflammatory disorders

**DOI:** 10.3389/fimmu.2023.1222776

**Published:** 2023-05-31

**Authors:** Maria Sole Chimenti

**Affiliations:** Rheumatology, Allergology and Clinical Immunology, System Medicine Department, University of Rome Tor Vergata, Rome, Italy

**Keywords:** women - diseases, women in academia, autoimmune disease (AD), autoinflammatory disease, women scientists

At present, less than 30% of researchers worldwide are women. Long-standing biases and gender stereotypes are discouraging girls and women away from science-related fields, and from research in particular. Science and gender equality needs worldwide attention and knowledge on unmet-needs for gender equality should be supported. This Research Topic is aimed at promoting the work of women scientists; in the papers considered for this collection, the first or last author is a woman researcher working across all fields related to autoimmune and autoinflammatory disorders. Despite the increasing attention on the issue of gender inequality, efforts are still needed to achieve gender parity in rheumatology. Considering this, women may be more sensitive to the evaluation and characterization of women affected by autoimmune diseases, in particular for pregnant women or women of childbearing-age. The papers presented in the Research Topic are related to autoimmune and autoinflammatory diseases and are authored by women scientists. The diversity of research performed across the Research Topic reflected the heterogenicity of autoimmune and autoinflammatory disorders and presents advances in pathogenesis and clinical and therapeutical patterns with applications for compelling problems. In the general population, women have a higher prevalence of autoimmune diseases with respect to men. Both organ-specific (such as autoimmune thyroiditis) and systemic autoimmune diseases are dominant in women (1). As is well reported in the literature, for example, the female-to-male ratio of systemic lupus erythematosus (SLE) prevalence is 9:1. SLE typically manifests between the second and fourth decade of life, affecting women of childbearing age (2). During pregnancy, SLE is associated with a high risk of perinatal morbidity and mortality, with frequent complications for both the mother and the fetus. As an example of autoinflammatory diseases, Takayasu arteritis, a form of systemic vasculitis, has a female-to-male ratio of 3.1:1. The disease is characterized by vascular inflammation and organ damage *via* vascular-inflammation, the prevalence is related to sex, and sex hormones may have a role in the pathogenesis. Inflammatory processes related to vascular damage may have key biomarkers and pathways that correlate with infiltrating immune cells (3). Hypotheses have been made for intracranial aneurysms (IA), where females have a significantly higher incidence of IA than males, with evidence that female sex steroids such as estrogen may play an important role in IA onset. Estrogen can enhance immune responses, resulting in many acquired immunity and autoimmune diseases. Moreover, recent advances have highlighted pathways related to placental dysfunction during pregnancy in women with autoimmune diseases playing a crucial role in understanding the pathogenesis of the diseases and the therapeutical approaches. The association between autoimmune diseases is particularly frequent in women. Pregnancy failure in women affected by systemic sclerosis consists mainly of preterm delivery, intrauterine growth restriction (IUGR), and very low birth weight, and thyroid dysfunctions and autoimmune thyroid disease, such as Hashimoto’s thyroiditis, represent a common feature of systemic sclerosis (SSc) (4). Since thyroid dysfunctions have been associated with pregnancy failure, in particular with recurrent spontaneous abortion (RSA), their presence in women with SSc can affect the pregnancy outcome (5). Last but not least, in the group of diseases known as spondylarthritis and defined as autoinflammatory or autoimmune diseases, peripheral and axial manifestations have different phenotypes and prevalence in men and women. They should not be seen as predominantly male diseases, as the non-radiographic form occurs with roughly equal frequency in women and men. However, men and women experience this disease differently (6). Furthermore, women tend to have less adherence and a lower response to treatment, so more gender-oriented data are needed regarding the drugs used for Axial-Spondyloarthritis (axSpA), especially biological disease-modifying antirheumatic drugs. Taken together, the hypothesis, evidence based-medicine, and new pathogenetic mechanisms suggests a complex interplay among sex hormones and immune system activation. With this Research Topic, we wanted to highlight the scientific relevance of women in science with the publication of papers and reviews related to autoimmune or anti-inflammatory diseases. Strengthening this association could contribute to developing therapeutic options and improving patient management, thus reducing the occurrence of worse consequences in disease activity and pregnancy outcomes.

## Author contributions

The author confirms being the sole contributor of this work and has approved it for publication.
